# Ligand Functionalization and Fe Precursor Selection Modulated the Physicochemical Properties of Fe-MOFs and Their Biological Response on Prostate Cancer and Normal Prostate Cells

**DOI:** 10.1021/acsomega.6c05924

**Published:** 2026-09-15

**Authors:** Zehra Durmus, Gizem Kugu, Zihni Onur Çalışkaner, Roberto Köferstein, Titus Lindenberg, A. Wouter Maijenburg

**Affiliations:** † Center for Innovation Competence (ZIK) SiLi-nano, 9176 Martin Luther University Halle-Wittenberg, Halle (Saale) 06120, Germany; ‡ 52962 Gebze Technical University, Department of Molecular Biology and Genetics, Gebze, Kocaeli 41400, Türkiye; § Department of Molecular Biology and Genetics, Faculty of Engineering and Natural Sciences, Biruni University, Istanbul 34015, Türkiye; ∥ Biruni University Advanced Technology Application and Research Center (B@MER), Biruni University, Istanbul 34015, Türkiye; ⊥ Inorganic Chemistry, Institute of Chemistry, Martin Luther University Halle-Wittenberg, Halle (Saale) 06120, Germany

## Abstract

Metal-organic frameworks (MOFs) offer modular platforms for cancer nanomedicine, but how metal precursor chemistry and linker functionalization jointly regulate physicochemical properties and biological response remains insufficiently defined. Here, three Fe-based MOFs, Fe-BDC-1, Fe-BDC-2, and Fe-NH_2_-BDC, were synthesized by solvothermal routes using BDC or NH_2_-BDC linkers and Fe nitrate or Fe chloride precursors. XRD, FTIR, SEM, magnetic measurements, UV–Vis diffuse reflectance, and photoluminescence analyses showed that Fe-BDC-1 and Fe-BDC-2 form closely related Fe-BDC frameworks, with FeCl_3_ promoting higher crystallinity and distinct particle assembly, whereas amino functionalization yields a structurally and optically distinct Fe-NH_2_-BDC phase with broadened visible-light absorption, lower band-gap energy, and enhanced emission. Biological activity was assessed in PC-3 prostate cancer cells and PNT1A normal prostate epithelial cells using WST-1 assays under magnet-free and magnet-exposed conditions, followed by DOX-combination testing and Annexin V/PI flow cytometry. Fe-BDC-2 displayed pronounced intrinsic toxicity toward PC-3 cells, including near-complete viability loss at 150 μg/mL after 48 h, while PNT1A cells showed comparatively lower sensitivity under selected conditions. Magnetic exposure further amplified cytotoxicity, particularly at higher doses and prolonged exposure. In WST-1 assays, Fe-NH_2_-BDC co-treatment with DOX produced the most consistent reduction in PC-3 metabolic viability under selected conditions. However, Annexin V/PI analysis did not indicate enhanced apoptosis and instead revealed predominantly PI-positive membrane damage with mixed cell-death features. Magnetic exposure further amplified cytotoxicity, particularly at higher doses and prolonged exposure. In WST-1 assays, Fe-NH_2_-BDC co-treatment with DOXorubicin (DOX) produced the most consistent reduction in PC-3 metabolic viability under selected conditions. However, Annexin V/PI analysis did not indicate enhanced apoptosis and instead revealed predominantly PI-positive membrane damage with mixed cell-death features. These findings identify linker functionalization and precursor selection as coupled design variables for tuning Fe-MOF structure, magnetoresponsive behavior, and chemotherapy-associated biological response. These findings identify linker functionalization and precursor selection as coupled design variables for tuning Fe-MOF structure, magnetoresponsive behavior, and chemotherapy-associated biological response.

## Graphical abstract

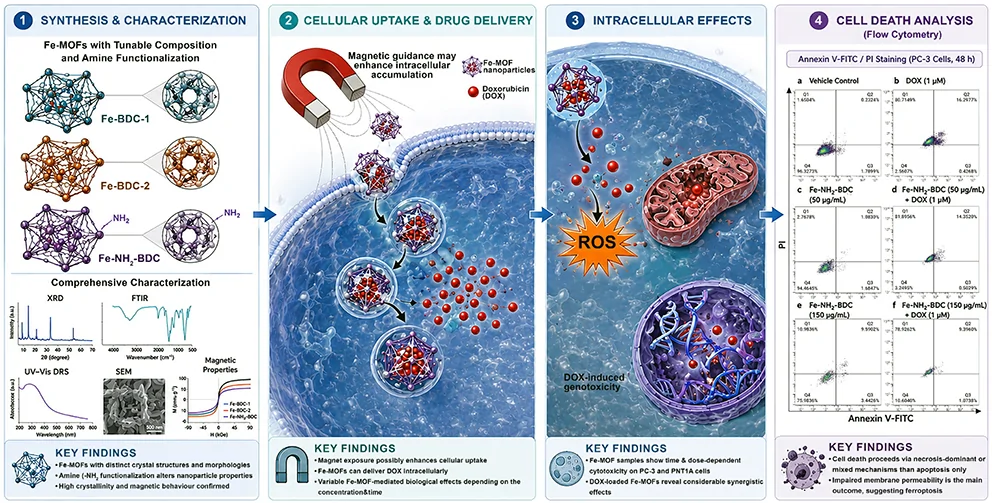

## 1 Introduction

Cancer remains one of the major global causes of morbidity and mortality, and its treatment is still constrained by limited tumor selectivity, systemic toxicity, drug resistance, inefficient penetration into solid tumors, and dose-limiting adverse effects. These limitations have sustained strong interest in nanoscale therapeutic platforms that can improve drug accumulation, protect labile cargoes, regulate release kinetics, and combine therapeutic and diagnostic functions within a single material system. In cancer nanomedicine, inorganic and hybrid nanomaterials are particularly attractive because their size, morphology, surface chemistry, degradation behavior, and stimulus responsiveness can be engineered to interact with the tumor microenvironment more selectively than many conventional molecular formulations.^1,2^

Within this broad nanomaterial landscape, metal-organic frameworks (MOFs) have emerged as highly tunable crystalline porous materials for cancer-related applications. MOFs consist of metal ions or clusters connected by multidentate organic linkers, giving rise to large surface areas, ordered porosity, modular compositions, and chemically addressable internal and external surfaces. These features allow MOFs to serve not only as carriers for chemotherapeutics, proteins, nucleic acids, photosensitizers, and other functional agents, but also as active components in stimuli-responsive and multimodal treatment schemes. Representative ZIF, UiO, MIL, PCN, IRMOF, porphyrinic, hafnium, zirconium, zinc, copper, and iron-containing MOF systems have been explored for chemotherapy, photodynamic therapy, photothermal therapy, chemodynamic therapy, radiosensitization, biosensing, biomarker detection, cancer cell typing, and theranostic applications.^2–10,10–13^

The current state of the art shows that MOFs have moved well beyond passive drug encapsulation. They can be designed to respond to acidic pH, reDOX gradients, ATP levels, enzymes, light, ultrasound, magnetic fields, or other internal and external triggers, thereby enabling site-preferential cargo release or activation of therapeutic mechanisms. In parallel, several MOF families have been adapted for biomarker detection and imaging-guided therapy, demonstrating that these frameworks can support both therapeutic intervention and analytical monitoring. However, despite extensive preclinical progress, clinical translation remains limited. Key unresolved issues include scalable and reproducible synthesis, batch-to-batch consistency, colloidal and physiological stability, premature degradation in biological media, protein corona formation, biodistribution, pharmacokinetics, immune response, long-term biodegradation, and comprehensive toxicity assessment. These challenges indicate that MOF performance must be interpreted as a structure-dependent biological response rather than a simple consequence of free metal ions or linker chemistry.^3–7^

Although Fe-containing MOFs and Fe-composited systems have also been reported as MRI-active agents and imaging-guided theranostic carriers, these functions are mentioned here only to position Fe-MOFs within the wider biomedical field and are not the central focus of the present study.^14–17^ Similarly, Fe-based composites have been incorporated into multimodal imaging, photothermal, photodynamic, radiosensitizing, and immunomodulatory strategies, but those directions are treated only as contextual evidence of platform versatility rather than as a detailed comparative section in this introduction.^13,18–27^

Fe-based MOFs are especially relevant in oncology because iron nodes can introduce reDOX activity, magnetic responsiveness, and biodegradability while preserving the porosity and compositional flexibility of the framework. Their Fe^2+^/Fe^3+^ chemistry can support Fenton or Fenton-like reactions in tumor-relevant environments, leading to reactive oxygen species generation, oxidative stress, chemodynamic effects, and ferroptosis-associated pathways. In addition, the coordination bonds between iron centers and carboxylate linkers can be destabilized under acidic, reductive, or metabolite-rich intracellular conditions, providing a basis for tumor-microenvironment-responsive degradation and cargo release. Accordingly, recent Fe-MOF systems based on MIL-100­(Fe), MIL-101­(Fe), MIL-53­(Fe), PCN-224-derived structures, polydopamine-coated Fe-MOFs, and related hybrids have been used to deliver DOX, quercetin, camptothecin, docetaxel, gemcitabine, tomentosin fractions, photosensitizers, enzymes, and nanozyme components in different cancer models.^28–35^

For the present work, the most relevant design question is not whether Fe-MOFs can support every possible theranostic modality, but how changes in iron precursor chemistry and linker functionalization alter the physicochemical properties of Fe-MOFs and their subsequent biological response. This question is important because parameters such as crystallinity, particle assembly, surface chemistry, optical behavior, and magnetic response can influence cell-material interactions, cytotoxicity, and drug-combination behavior. Amino functionalization is particularly important because it can modify electronic structure, hydrogen-bonding capacity, surface polarity, and potential interactions with drug molecules or biological interfaces, whereas the iron precursor can affect framework formation, particle morphology, crystallinity, and residual coordination environment.

In this study, three Fe-based MOFs, Fe-BDC-1, Fe-BDC-2, and Fe-NH2-BDC, were synthesized by solvothermal routes to examine the coupled influence of Fe precursor selection and linker functionalization. Fe-BDC-1 and Fe-BDC-2 were prepared using 1,4-benzenedicarboxylic acid with different iron sources, while Fe-NH2-BDC was synthesized using 2-aminoterephthalic acid to introduce amino functionality. The materials were characterized by XRD, FTIR, SEM, UV-Vis diffuse reflectance spectroscopy, photoluminescence spectroscopy, and magnetic measurements. Their biological responses were then evaluated in PC-3 prostate carcinoma cells and PNT1A normal prostate epithelial cells under magnet-free and magnet-exposed conditions, followed by DOX-combination testing and Annexin V/PI flow cytometry. This approach links synthetic design variables with structure, optical and magnetic properties, intrinsic cytotoxicity, and chemotherapy-enhancing behavior in prostate cell models.

## 2 Experimental Details

### 2.1 Materials

Terephthalic acid (1,4-BDC), 2-aminoterephthalic acid (NH_2_-BDC), iron­(III) nitrate nonahydrate (Fe­(NO_3_)_3_·9H_2_O), iron­(III) chloride hexahydrate (FeCl_3_·6H_2_O), and N,N-dimethylformamide (DMF) were purchased from Carl Roth and Sigma-Aldrich. All chemical reagents were of analytical grade and used as received without any further purification. Deionized (DI) water was used throughout the experiments.

### 2.2 Synthesis of Fe-MOFs

The Fe-MOF variants were synthesized using a uniform solvothermal approach to evaluate the impact of different metal precursors and organic linkers on the final framework structure, as depicted in Figure 1.

**Figure 1:**
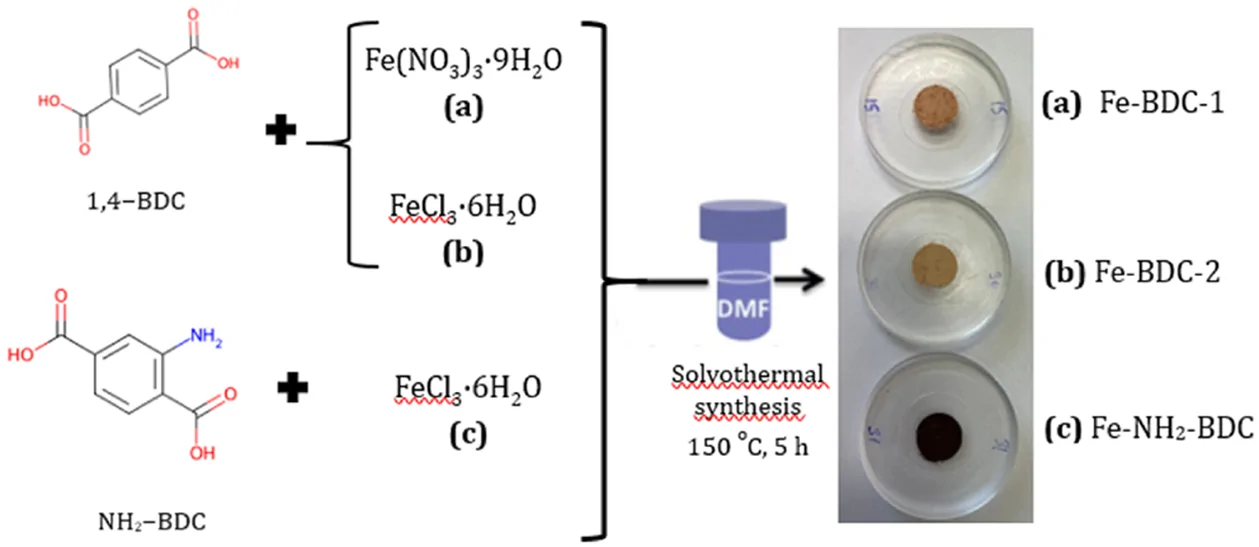
The Schematic illustration for the preparation of **a)** Fe-BDC-1, **b)** Fe-BDC-2, and **c)** Fe-NH_2_-BDC. Photograph courtesy of “Zehra Durmus”. Copyright 2025.

### 2.3 Synthesis of Fe-BDC-1

In a typical synthesis, ferric nitrate nonahydrate (Fe­(NO_3_)_3_·9H_2_O) and 1,4-BDC were dissolved in N,N-dimethylformamide (DMF) (molar ratio: 1:1:280). After ultrasonication for 15 min, the reaction mixture was transferred into a Teflon-lined stainless steel autoclave. The autoclave was heated from room temperature to 150 °C at a rate of 5 °C min^–^ 1 and maintained at this temperature for 5 h. After completion of the reaction, the autoclave was allowed to cool naturally to room temperature in the oven. The resulting light orange-brown precipitate was collected via centrifugation, washed thoroughly with DMF and ethanol several times to remove unreacted precursors, and dried under vacuum at 60 °C for 5 h.

### 2.4 Synthesis of Fe-BDC-2

The procedure was identical to the synthesis of Fe-BDC-1, with the exception that iron­(III) chloride (FeCl_3_·6H_2_O) was used as the metal precursor instead of iron­(III) nitrate. The final product was collected as a yellow-brown powder.

### 2.5 Synthesis of Fe-NH_2_-BDC

To introduce amine functional groups into the framework, NH_2_-BDC was utilized as the organic linker alongside iron­(III) chloride (FeCl_3_·6H_2_O). The dark blackish-brown product was isolated, washed, and dried following the aforementioned purification protocol.

### 2.6 Characterization Studies

The structural properties of the obtained samples were determined by X-ray diffractometry (XRD) (Bruker D8 Advance, Cu–K_α_, λ = 0.15406 nm, 40 kV, 40 mA) in the 2θ range of 5°-70° with a constant shutter speed, a time-step of 0.5, and a step size of ca. 0.01°. The software DiffracPlus EVA (Bruker) was used for data evaluation and background correction. The chemical structure of the obtained samples was characterized with/by Fourier-Transform infrared spectroscopy (FTIR, Tensor 27, Bruker Co.) equipped with a diamond-ATR unit in the wavenumber range of 4000–400 cm^–1^. The morphological properties of the samples were characterized by a scanning electron microscope (SEM, Carl Zeiss Merlin). The UV–Vis diffuse reflection spectra (DRS) of the samples were recorded by a UV-Vis diffuse reflectance spectrometer (Agilent, Cary 60) in the wavelength range of 200–800 nm. BaSO_4_ was used as the substrate and the obtained reflectance spectra were converted to absorbance by the Kubelka–Munk method. Magnetic measurements were carried out using a Quantum Design PPMS 9 system. Temperature-dependent investigations were carried out at H_DC_ = 6 kOe under field-cooled (FC) conditions between 5 and 300 K. Additionally, field-dependent measurements were taken at 300 and 5 K with magnetic DC field cycling between ±90 kOe.

### 2.7 Cell Culture and Preparation of Fe-MOF and DOX Solutions

Prostate Cancer-3 (ATCC, #CRL-1435) and Prostate Normal Tissue 1A (Cellosaurus, #CVCL-2163) were cultured in high-glucose Dulbecco’s Modified Eagle Medium (Thermo Fisher, USA), supplemented with 10% (v/v) fetal bovine serum and 1% (v/v) penicillin/streptomycin (10,000 U/mL), in a humidified incubator (37 °C and 5% CO_2_). Cells were maintained at 70% confluence by passaging once every 3 days on average.

Each fabricated Fe-MOF powder was dissolved in sterile dimethyl sulfoxide (DMSO) at a concentration of 10 mg/mL to prepare a master stock solution, followed by vortexing to ensure complete dispersion. Before cell treatment, Fe-MOF stock solutions were diluted in DMEM to obtain the desired working concentrations. DOX stock solution was prepared at 10 μM using ultrapure DNase/RNase-free distilled water. For cell treatments, DOX was diluted in culture medium to achieve a final concentration of 1 μM in each well.

### 2.8 Cell Viability Assay

All experiments were performed as three independent biological repeats, each including three technical replicates (triplicates). Cell viability was assessed using the WST-1 cell proliferation reagent (Sigma-Aldrich, 11644807001) according to the manufacturer’s instructions.

Cells were seeded in 96-well plates (100 μL/well) 1 day before treatment to achieve approximately 60–70% confluence at the time of drug application. Therefore, PC-3 cells were seeded at a density of 1.1 × 10^4^ cells/well, while PNT1A cells were seeded at 1.7 × 10^4^ cells/well. Following overnight incubation under standard culture conditions, treatment groups were established as follows: DMEM only (untreated control), DMEM + DMSO (vehicle control), DMEM containing each Fe-MOF, DMEM containing DOX, and DMEM containing a combination of Fe-MOF and DOX. Fe-MOF treatments were applied at final concentrations of 50 μg/mL and 150 μg/mL, while DOX was administered at a final concentration of 1 μM in all relevant wells. For combination treatments, Fe-MOF and DOX were co-administered at the same concentrations to evaluate potential synergistic effects on cell viability.

Cell viability was assessed 2 h (day zero) and 48 h post-treatment using the WST-1 assay. Following incubation with the WST-1 reagent, absorbance was measured at 440 nm with a reference wavelength of 660 nm using a microplate reader. Absorbance values of the triplicates were first averaged, and the averaged value of only the culture medium (blank) was subtracted from all measurements. Considering the intrinsic absorbance potential of Fe-MOFs at the designated wavelength, additional control/blank wells for each compound (DMEM containing each Fe-MOF sample with varying concentrations, 50 μg/mL and 150 μg/mL, without cells) were prepared and measured under identical conditions. After blank normalization, the absorbance values obtained from these cell-free Fe-MOF-containing wells were subtracted from the corresponding Fe-MOF-treated wells to eliminate material-derived signal interference. Relative cell viability was then calculated by normalizing the corrected absorbance values of treated groups to the untreated control (vehicle). Growth curves were represented based on these normalized % viability values. All experiments were independently repeated three times.

### 2.9 Cell Viability Assay Under Magnetic Exposure

A cell viability assay was also conducted to estimate the effects of the magnetic field on the Fe-MOF action. Thus, two identical 96-well plates were prepared as stated above. One plate was incubated without magnetic field exposure (magnet -), while the second plate was subjected to an external magnetic field (magnet +). The magnetic field was applied using a strong neodymium (NdFeB) magnet placed directly beneath the culture plate at a fixed distance. Cells were incubated at 37 °C in a humidified atmosphere with 5% CO_2_ throughout the experiment. To introduce temporal variation in the magnetic stimulus, the orientation of the magnet was manually reversed every 12 h during the incubation period. At the specified time points, the WST-1 assay was implemented as elaborated above.

### 2.10 Apoptosis Assay by Flow Cytometry

Apoptosis analysis was performed in PC-3 cells using flow cytometry. Cells were seeded in 48-well plates at a density of approximately 3 × 10^4^ cells/well to achieve 60–70% confluence at the time of treatment. Each experimental condition was prepared in duplicate wells. Following overnight incubation at 37 °C in a humidified atmosphere with 5% CO_2_, cells were treated with the following conditions: DMEM only (control), DMEM containing Fe-MOF, DMEM containing DOX, and DMEM containing a combination of Fe-MOF and DOX. Fe-MOF treatments were applied at different final concentrations of 50 μg/mL and 150 μg/mL, while DOX was used at a final concentration of 1 μM. For combination treatments, Fe-MOF and DOX were co-administered at the same concentrations.

After 48 h of treatment, cells from duplicate wells corresponding to the same condition were harvested and pooled before apoptosis analysis. Subsequently, apoptosis was evaluated using the Annexin V-FITC Apoptosis Detection Kit (eBioscience, #BMS500FI-20), following the manufacturer’s protocol. Briefly, pooled cells were washed with cold phosphate-buffered saline (PBS) and resuspended in ice-cooled binding buffer. Cells were then stained with Annexin V-FITC and propidium iodide (PI) according to the kit instructions. Stained cells were subsequently analyzed by the Beckman Coulter Cytoflex flow cytometry system to quantify cell populations, acquiring 20000 ungated events. Data were evaluated to distinguish viable (Annexin V^–^ /PI^–^), early apoptotic (Annexin V^+^/PI^–^), late apoptotic (Annexin V^+^ /PI^+^), and necrotic (Annexin V^–^ /PI^+^) cell fractions utilizing FlowJo v11 software.

### 2.11 Statistical Analysis

Two-way ANOVA was used for the multiple statistical comparisons of the groups (samples, doses, times, magnetic conditions, etc.) by utilizing GraphPad Prism 9.5.0 software. Additionally, graphs were also plotted by the same software.

## 3 Results and Discussion

### 3.1 Functional, Structural, and Morphological Characterization

The FTIR spectra provide additional evidence for the successful formation of Fe-carboxylate frameworks, and they are in good agreement with the XRD results shown in Figure 2. The spectra of Fe-BDC-1 and Fe-BDC-2 are highly similar, indicating that both samples possess a closely related coordination environment and most likely belong to the same Fe-BDC framework family. In both materials, the disappearance of the characteristic free carboxylic acid C=O stretching band expected near 1700–1720 cm^–1^ indicates deprotonation of the linker during synthesis. Instead, two prominent bands are observed at approximately 1580–1600 cm^–1^ and 1380–1420 cm^–1^, which can be assigned to the asymmetric and symmetric stretching vibrations of coordinated carboxylate groups, ν_as_(COO^–^) and ν_s_(COO^–^), respectively. These features confirm that the BDC ligand is coordinated to Fe centers in deprotonated form. Additional absorptions in the 750–500 cm^–1^ region can be attributed to aromatic ring deformation modes together with possible Fe–O vibrations, further supporting the formation of an Fe-organic coordination framework.

**Figure 2:**
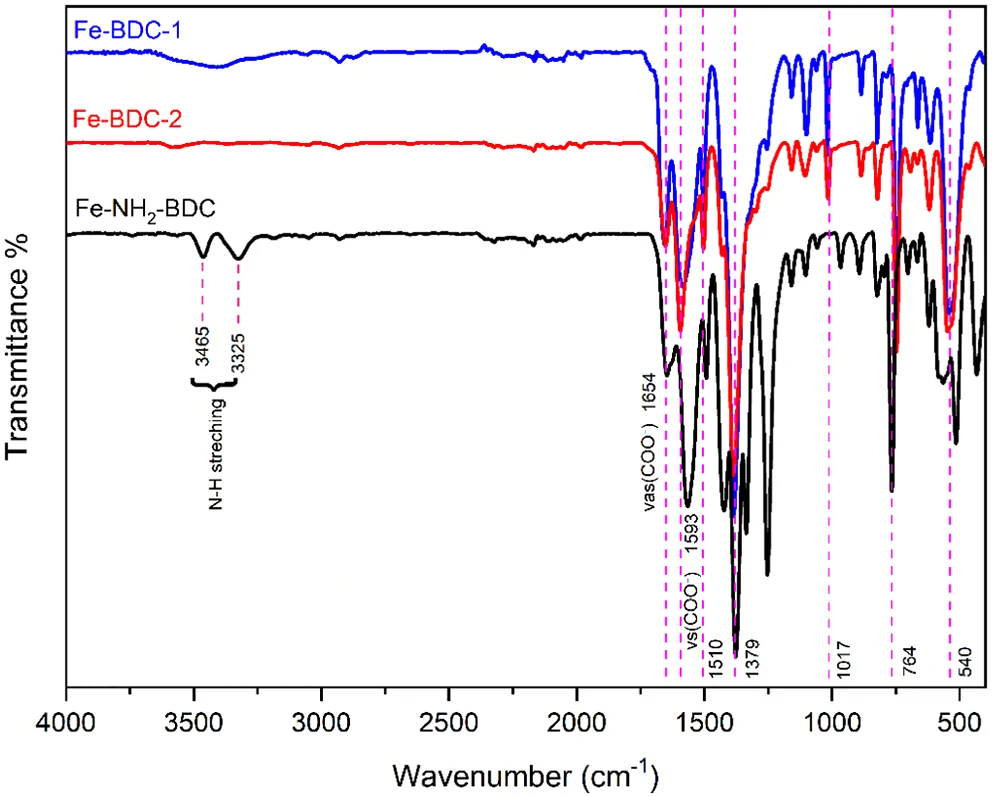
FTIR spectra of Fe-BDC-1, Fe-BDC-2, and Fe-NH_2_-BDC samples.

By contrast, the Fe-NH_2_-BDC sample exhibits a distinctly different FTIR profile, confirming the effect of ligand functionalization on the final structure. The most notable difference is the appearance of additional bands in the 3500–3300 cm^–1^ region, which are assigned to the N–H stretching vibrations of the amino group, demonstrating that the amine functionality is preserved after synthesis. Moreover, the spectral region around 1600–1500 cm^–1^ differs substantially from that of Fe-BDC-1 and Fe-BDC-2, likely due to overlapping contributions from ν_as_ (COO^–^), aromatic C=C vibrations, and N–H bending modes of the amino-substituted linker. Changes throughout the fingerprint region below 1500 cm^–1^ also indicate a modified local coordination environment in comparison with the non-functionalized BDC-based materials. Therefore, the FTIR data support the conclusion that Fe-BDC-1 and Fe-BDC-2 are chemically very similar and likely belong to the same structural family, whereas Fe-NH_2_-BDC represents a distinct amino-functionalized Fe-MOF phase.

The crystalline structures and phase purity of the synthesized Fe-BDC-1, Fe-BDC-2, and Fe-NH_2_-BDC samples were evaluated using X-ray diffraction (XRD), as illustrated in Figure 3. Since metal-organic frameworks (MOFs) are typically characterized via single-crystal data and lack established standard JCPDS/ICDD card numbers, the experimental diffraction profiles were indexed and compared using the corresponding Cambridge Structural Database (CSD) references.

**Figure 3:**
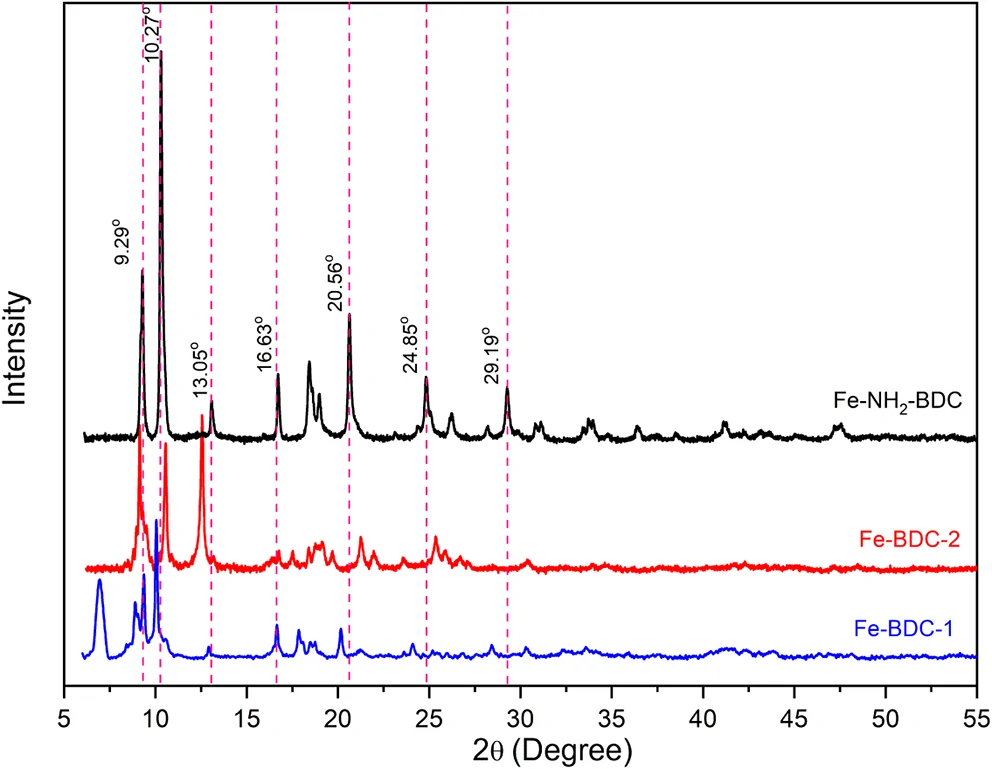
XRD patterns of Fe-BDC-1, Fe-BDC-2, and Fe-NH_2_-BDC samples.

The XRD results indicate that samples Fe-BDC-1 and Fe-BDC-2, both synthesized using 1,4-BDC as the organic linker, firmly match the simulated XRD pattern of the open-framework MIL-53­(Fe) topology (CSD 258444). The appearance of the main diffraction peaks in similar 2θ degree regions implies that changing the iron precursor from iron nitrate to iron chloride did not substantially alter the resulting framework topology under the applied synthesis conditions. However, compared to Fe-BDC-2, the pattern of Fe-BDC-1 shows two additional reflections at 2θ = 6.5° and 8.5°, which are attributed to the well-known framework flexibility (breathing effect) or structural variations of the MIL-53 topology containing trapped solvent molecules within the channels. Furthermore, the significantly sharper and more intense reflections observed for sample Fe-BDC-2 suggest a higher degree of crystallinity and a more ordered long-range framework structure when iron chloride was utilized as the metal source.

In contrast, sample Fe-NH_2_-BDC, synthesized using amino-BDC and iron chloride, displays a clearly different diffraction pattern compared with the non-functionalized MIL-53­(Fe) samples. This distinct variance indicates that the introduction of the amino functional group significantly influenced the self-assembly process, inducing a complete phase transformation toward the hexagonal MIL-88B­(Fe)-NH_2_ topology (CSD 647646). The diffraction profile of Fe-NH_2_-BDC features two prominent and sharp characteristic peaks at 2θ = 9.29° and 10.27°, which are indexed to the (002) and (101) crystal planes of the hexagonal MIL-88B phase, respectively. This phase alteration is directly verified by the steric and electronic effects introduced by the -NH_2_ functional groups on the BDC linker, which thermodynamically favor the assembly of the MIL-88B framework rather than the MIL-53 or MIL-101 topologies under the given synthetic conditions.

Furthermore, considering potential applications in biological media, iron-based frameworks such as MIL-53­(Fe) and MIL-88B­(Fe) are reported in the literature to possess excellent structural stability and integrity when in contact with aqueous solutions or cell culture media like DMEM.^36,37^

The SEM images reveal clear morphological differences among the synthesized samples (Figure 4). Fe-BDC-1 exhibits a morphology dominated by densely packed rod-like particles, which assemble into larger aggregated secondary structures, highly characteristic of the MIL-53­(Fe) phase. In contrast, Fe-BDC-2 shows a much more agglomerated and irregular granular morphology with less distinguishable individual crystals and a rougher surface texture. This difference suggests that although Fe-BDC-1 and Fe-BDC-2 may belong to the same MIL-53­(Fe) structural family, the change in iron precursor significantly influenced crystal growth behavior and particle assembly, where iron chloride yielded a higher structural density. On the other hand, Fe-NH_2_-BDC displays a clearly distinct morphology characterized by plate-like or petal-like particles arranged in flower-like aggregates. This unique morphology differs markedly from both non-functionalized BDC-based samples and serves as a morphological blueprint indicating that amino functionalization strongly affects nucleation and crystal growth, promoting a structural shift toward the MIL-88B­(Fe) architecture. Overall, the SEM observations support the structural characterization results, showing that Fe-BDC-1 and Fe-BDC-2 are structurally related within the MIL-53­(Fe) framework topology, whereas Fe-NH_2_-BDC forms a distinctly different amino-functionalized MIL-88B­(Fe) architecture.

**Figure 4:**
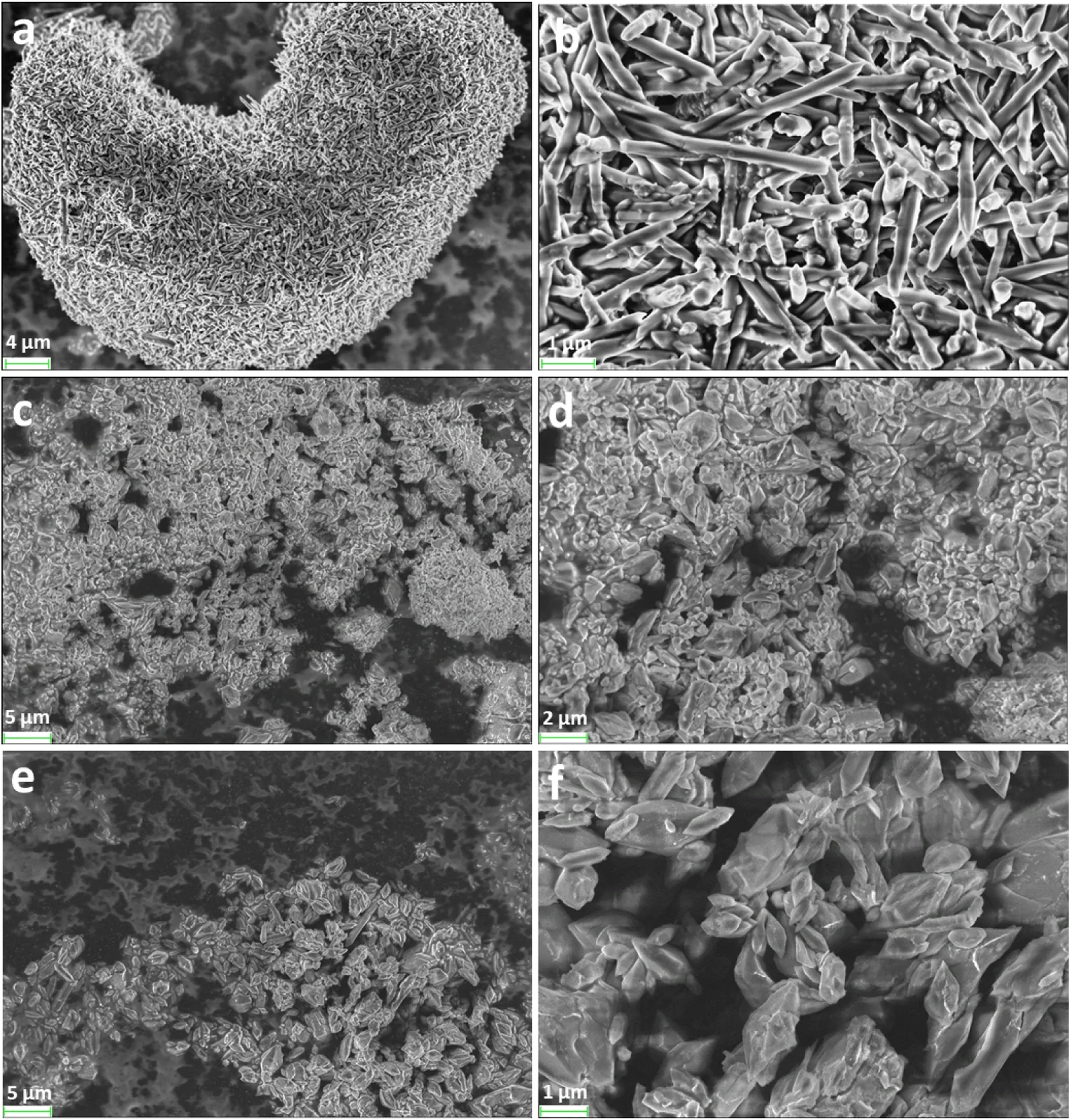
SEM images of **a, b)** Fe-BDC-1, **c, d)** Fe-BDC-2, and **e, f)** Fe-NH_2_-BDC samples.

Figure 5a shows the temperature development of the mass susceptibility (χ_g_) between 5 and 300 K. For all three Fe-MOFs, the susceptibility increases with decreasing temperature, typical for a paramagnetic behavior. The plot χ_g_T versus temperature (inset in Figure 5a) exhibits a continuous decrease of χ_g_T to lower temperatures, and an additionally more pronounced decrease of χ_g_^–1^ below about 80 K, suggesting a possible canted antiferromagnetic coupling between neighboring iron cations as described for e.g., MIL-53.^38^ Field-dependent measurements at 300 and 5 K show a linear increase of the magnetization with the applied field, and no hysteresis loop is observed, confirming the paramagnetic character. The field-dependent magnetization of Fe-MOFs follows a Brillouin function, resulting in a slight curvature at high magnetic fields and low temperatures (*k*_B_ *T* < μ_B_B) (inset in Figure 5b). The magnetization values of Fe-BDC-2 are slightly higher than those of Fe-BDC-1, resulting from differences in the formed frameworks as indicated by the XRD measurements. Moreover, the magnetization can also be influenced by defects and particle sizes. As mentioned above, the XRD pattern of Fe-BDC-2 indicates a higher crystallinity, resulting in fewer defects compared to Fe-BDC-1.

**Figure 5:**
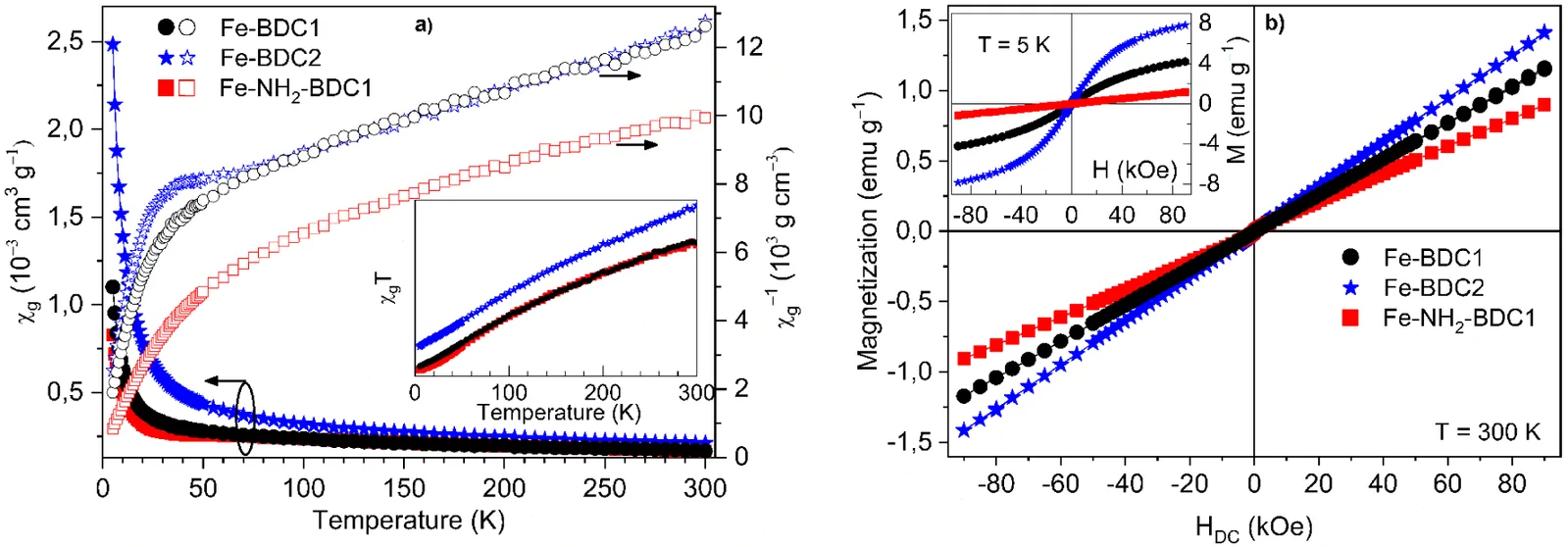
(a) Magnetic mass susceptibility (χ_g_) versus temperature under field-cooled conditions (H_DC_ = 6 kOe). The inset shows χ_g_T depending on temperature, and (b) evolution of the magnetization depending on the applied field (H_DC_) at 300 and 5 K.

The UV–Vis DRS spectra show that Fe-BDC-1 and Fe-BDC-2 have very similar optical responses, with strong absorption in the UV region and a weaker absorption tail extending into the visible region, indicating closely related electronic structures in Figure 6. In Fe-based MOFs, absorption bands in the 250–350 nm region are commonly assigned to ligand-to-metal charge transfer (LMCT) from carboxylate oxygen atoms to Fe centers, whereas the broader absorption in the 350–500 nm region is generally attributed to d–d transitions of Fe­(III) in the Fe–oxo environment in Figure 6a. Similar assignments have been reported for Fe-based MOFs, where the UV band is linked to charge transfer involving the carboxylate oxygen and the visible band to Fe­(III) transitions, and where Tauc analysis yields band-gap values in the visible-light-responsive range.^39,40^

**Figure 6:**
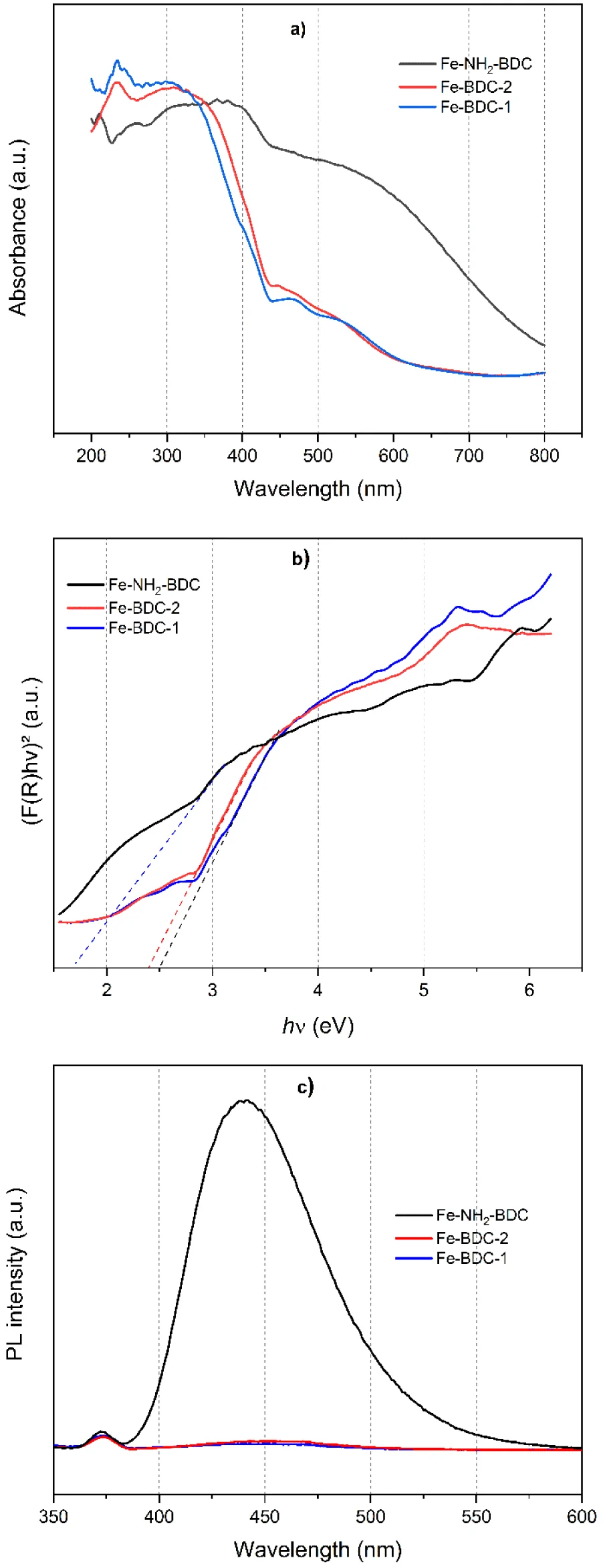
(a) UV–Vis DRS, (b) Tauc plots of the Fe-BDC-1, Fe-BDC-2, and Fe-NH_2_-BDC samples, and (c) photoluminescence spectra of Fe-BDC-1, Fe-BDC-2, and Fe-NH_2_-BDC samples recorded under 330 nm excitation.

By contrast, Fe-NH_2_-BDC exhibits a much broader and more intense absorption extending to longer wavelengths, which indicates that amino functionalization significantly modifies the electronic structure and improves visible-light harvesting. This trend is consistent with the general behavior of Fe-MOF systems, where stronger visible absorption and a red-shifted absorption edge are associated with a reduced optical band gap and improved utilization of visible light.^40,41^ The Tauc plots are also in agreement with this interpretation. Fe-BDC-1 and Fe-BDC-2 display nearly comparable band-gap energies, showing that changing the iron precursor did not substantially alter the band structure of the BDC-based framework, whereas Fe-NH_2_-BDC shows a distinctly lower band-gap value in Figure 6b. Therefore, the overall trend can be expressed as E_g_(Fe-NH_2_-BDC) < E_g_(Fe-BDC-1) ≈ E_g_(Fe-BDC-2). This result is important because reduced band-gap energy and altered electronic surface states in Fe-based MOFs are directly correlated with modifications in surface polarity, which govern the framework’s molecular interactions with chemotherapeutic agents like DOX and cell membrane interfaces. Taken together, the UV–Vis DRS and Tauc results indicate that Fe-BDC-1 and Fe-BDC-2 belong to the same or a closely related optical family, while Fe-NH_2_-BDC possesses a distinctly altered electronic structure due to amino functionalization, which is fully consistent with the XRD, FTIR, and SEM results.

The PL spectra in Figure 6c provide further evidence of the effect of ligand functionalization on the photophysical properties of the materials. Fe-NH_2_-BDC exhibited a strong and broad emission band centered at approximately 440–450 nm, whereas Fe-BDC-1 and Fe-BDC-2 showed only very weak emission in the same region, consistent with the reported photoluminescence behavior of amino-functionalized Fe-based MOFs.^42^ The emission maximum at approximately 440–450 nm corresponds to a photon energy of approximately 2.76–2.82 eV. Together with the red-shifted absorption edge and the lower Tauc-derived optical band gap of Fe-NH_2_-BDC, the PL response supports an amino-functionalization-induced modification of the electronic structure; however, direct numerical equivalence between the PL emission energy and the Tauc-derived band gap should not be assumed. This result indicates that amino functionalization substantially alters the excited-state electronic structure and enhances the luminescence response of the Fe-MOF. Beyond its potential use in fluorescence-based investigation, the altered electronic response and surface amino functionality of Fe-NH_2_-BDC provide a physicochemical rationale for evaluating this material in combination with DOX. Amino-functionalized MOFs have been investigated as DOX-delivery platforms because their surface chemistry can promote drug–framework interactions and can be further tailored for controlled or stimulus-responsive delivery.^43–45^ However, DOX loading, release, cellular uptake, and intracellular localization were not quantified in the present study; therefore, these findings do not establish an effective drug-delivery mechanism. In contrast, the very similar and weak PL signals of Fe-BDC-1 and Fe-BDC-2 suggest that changing the iron precursor has only a limited effect on the radiative relaxation behavior of the BDC-based materials. Overall, the PL findings are consistent with the UV-Vis DRS results and further confirm that linker functionalization is the dominant design variable governing the optical and electronic response of these Fe-based MOFs.

Taken together, the structural, morphological, and spectroscopic characterization results demonstrate that Fe-BDC-1 and Fe-BDC-2 are closely related MIL-53­(Fe) materials differing mainly in crystallinity and particle morphology, whereas Fe-NH_2_-BDC is a clearly distinct amino-functionalized MIL-88B­(Fe) matrix with modified crystal structure, morphology, and optical properties. These differences are expected to influence not only the physicochemical behavior of the materials but also their targeted therapeutic interactions in subsequent biological evaluations on prostate carcinoma (PC-3) and normal prostate (PNT1A) cells.

### 3.2 Cytotoxicity Profile of Fe-MOFs in Malignant and Nonmalignant Prostate Cell Lines

Initially, the intrinsic cytotoxic effects of the synthesized Fe-based metal–organic frameworks (Fe-BDC-1, Fe-BDC-2, and Fe-NH_2_-BDC) were evaluated in the malignant prostate cancer cell line (PC-3) in comparison with the nonmalignant prostate epithelial cell line (PNT1A) at two exposure times (2 h/time zero and 48 h/day 2) and concentrations (50 and 150 μg/mL). As illustrated in Figure 7, all frameworks exhibited pronounced time- and dose-dependent biological responses in the PC-3 and PNT1A cell lines, overall inducing a reduction in cell viability, which correlates with their physicochemical properties.

**Figure 7:**
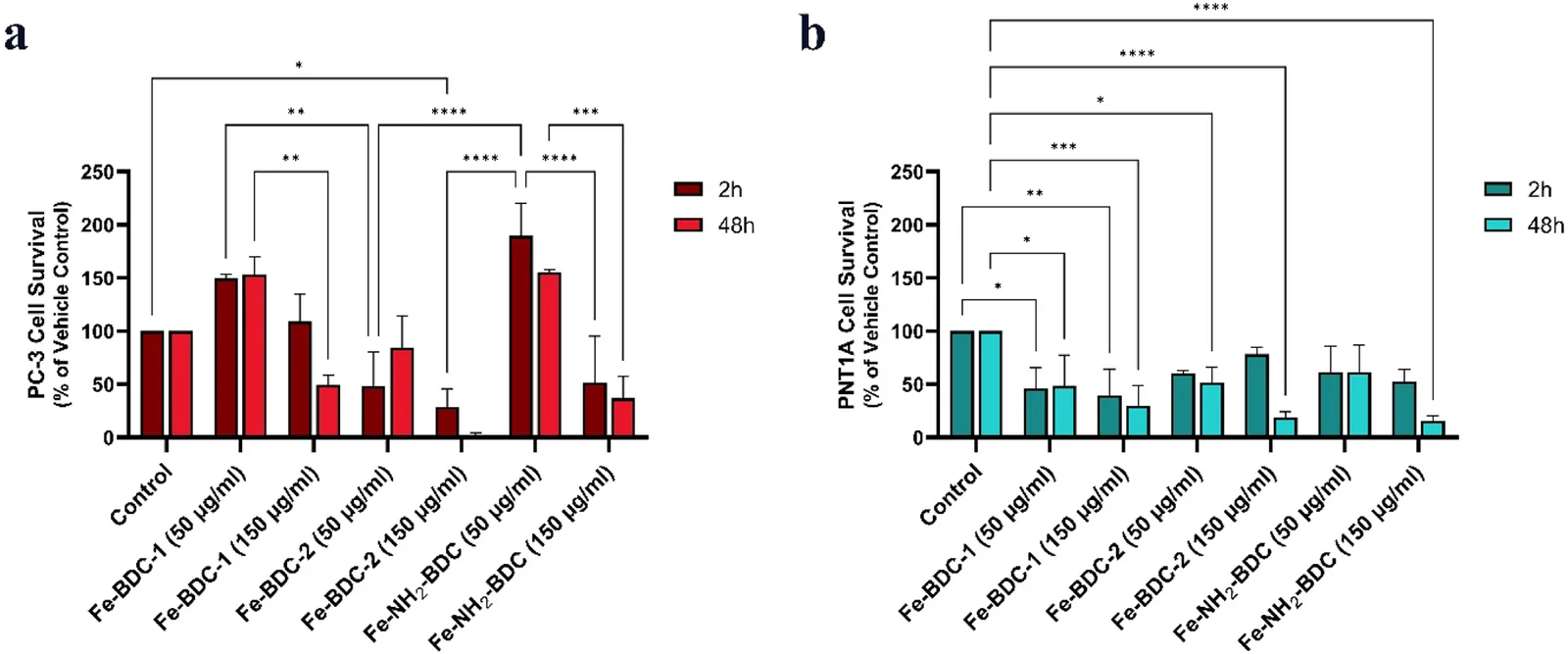
Comparison of the concentration- and time-dependent cytotoxic effects of the synthesized Fe-MOF samples on the (a) PC-3 and (b) PNT1A cell lines. Statistical analysis (mean ± SD, n = 3 independent experiments) was performed using two-way ANOVA (*p ≤ 0.05, **p ≤ 0.01, ***p ≤ 0.001, ****p ≤ 0.0001).

In PC-3 cells, short-term exposure (2 h) to Fe-BDC-1 and Fe-NH_2_-BDC interestingly caused no apparent reduction in cell viability at the lower concentration (50 μg/mL) (Figure 7a), suggesting a transient and rapid metabolic adaptation in these aggressive cancer cells. Similar transient increases in cell viability and biocompatible doses upon MOF administration have been reported in certain cancer cell lines.^46,47^ However, boosting the concentration to 150 μg/mL for Fe-BDC-1 and Fe-NH_2_-BDC resulted in a marked decline in the PC-3 survival relative to control over time. Notably, Fe-NH_2_-BDC represented a sharp 3–4-fold reduction (∼50% at 2 h and ∼35% at 48 h) in the viability between low- and high-dose conditions (Figure 7a). This finding suggests that amine functionalization might have contributed to cellular uptake and cytotoxic potential, likely through improved surface interactions.^43,48^ Otherwise, Fe-BDC-2 showed a more prominent cytotoxic profile at all concentrations and durations. At the higher concentration (150 μg/mL), it diminished the PC-3 viability by roundly 70% and 100% compared to the control at 2 and 48 h, respectively (Figure 7a).

Unlike PC-3 cells, all Fe-MOF constructs displayed more straightforward and plausible cytotoxicity patterns in the nonmalignant PNT1A control cell line at both concentrations and time points. All frameworks averagely revealed a decline in PNT1A viability by half relative to the vehicle control at the initial time point (2 h) without furthering a considerable deterioration at 48 h (Figure 7). Intriguingly, Fe-BDC-2 at 150 μg/mL has been relatively detected as less cytotoxic in PNT1A at both early and later stages (Figure 7b) compared to the malignant PC-3 cell line (Figure 7a), probably indicating a moderate and selective resistance in nonmalignant prostate cells.

These data altogether disclose that the malignant PC-3 cells and the nonmalignant PNT1A cells exhibited a concentration- and time-dependent sensitivity against the fabricated FeMOF subtypes. Particularly, Fe-BDC-2 markedly possessed more intrinsic toxicity toward prostate cancer cells than the normal prostate epithelial cells (Figure 7).

### 3.3 Effects of the Magnetic Field on the Fe-MOF Cytotoxicity

Next, experiments were conducted near a magnet (+) and magnet-free (−) conditions to determine whether an external magnetic field modulates MOF-induced cytotoxicity. Overall, magnetic exposure appears to influence FeMOF-mediated effects in both cell lines (Figure 8).

**Figure 8:**
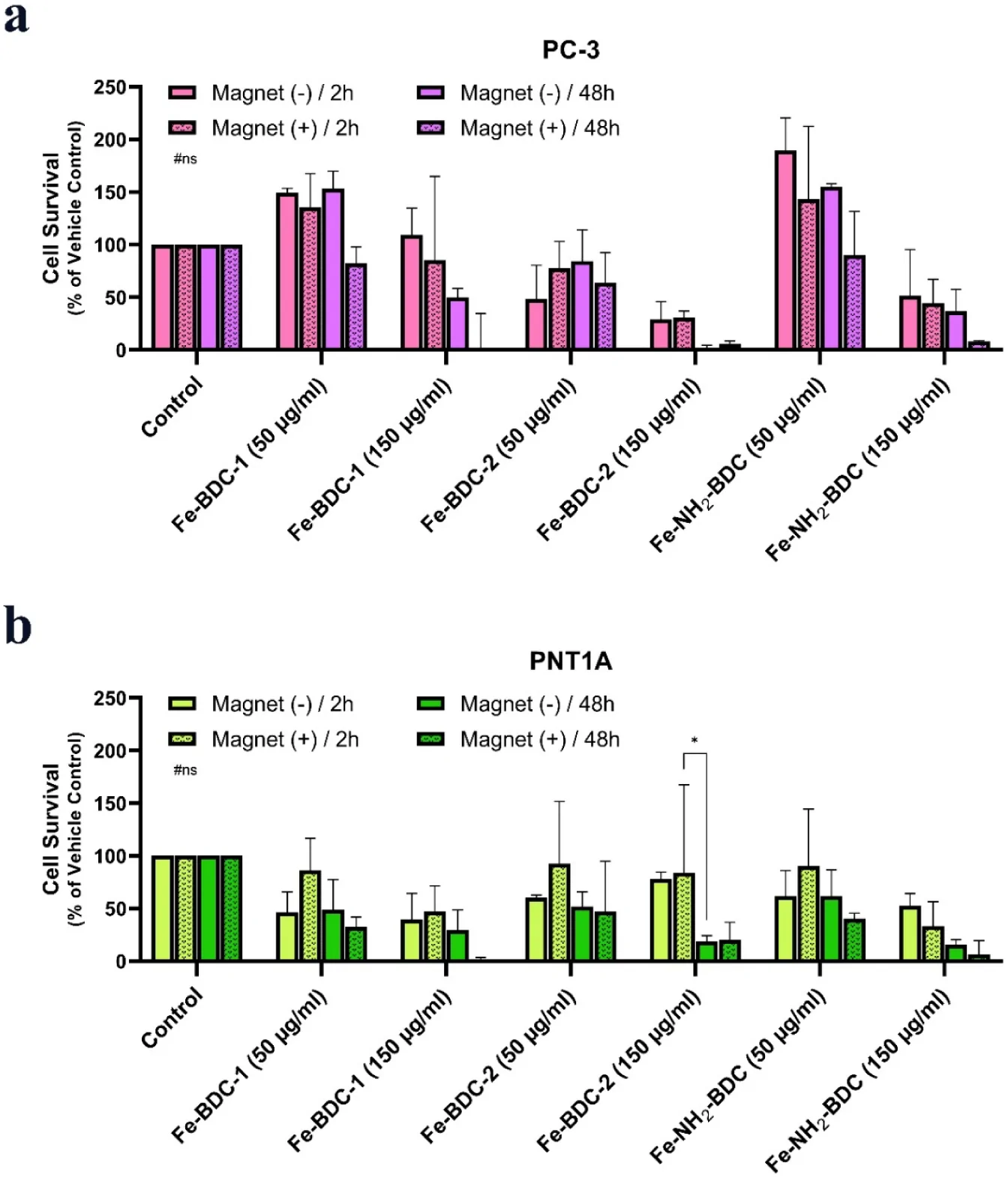
Magnet-influenced cytotoxicity of Fe-MOFs on the (a) PC-3 and (b) PNT1A cell lines. Statistical analysis (mean ± SD, n = 3 independent experiments) was performed using two-way ANOVA (*p ≤ 0.05, #statistically non-significant).

Magnet stimulation consistently enhanced cytotoxicity in PC-3 cells, especially at higher concentrations and/or longer administration times (Figure 8). For instance, exposure to Fe-BDC-1 at 50 μg/mL for 2 h under a magnetic field had no obvious effect on the cell viability, whereas the same dose under the magnet (+) condition resulted in a 1.9-fold decrease in the cell viability according to the magnet (−) group (Figure 8a). Moreover, administration of the higher concentration of Fe-BDC-1 (150 μg/mL) for 48 h under the magnet (+) furthered the cytotoxicity by drastically reducing PC-3 survival to nearly zero, indicating near-complete cytotoxicity relative to the magnet-free group. Consequently, Fe-BDC-2 induced stronger cytotoxicity in PC-3 cells than Fe-BDC-1 under both magnetic conditions. This enhanced activity can be associated with Fe-BDC-2’s comparatively higher crystallinity in XRD output (Figure 3) and slightly higher magnetization (Figure 6). Likewise, Fe-NH_2_-BDC also exhibited a dramatic magnet-enhanced response, reducing PC-3 viability by about 1.7- and 5.1-fold at 50 μg/mL and 150 μg/mL, respectively, at 48 h (Figure 8a).

Magnetic effects were also evident in PNT1A cells. Fe-BDC-1 at the higher concentration across 48 h again showed the strongest response in the presence of a magnet with approximately a complete eradication (Figure 8b). However, 50 μg/mL and 150 μg/mL of Fe-NH_2_-BDC under the magnet (+) condition caused only 1.5- and 2.3-fold decreases in PNT1A viability compared to the magnet (−) state, respectively. Magnetic field-improved cytotoxicity has been widely reported for iron-containing metal-organic-frameworks.^49–52^ Corresponding to these studies, a magnetic field can promote nanoparticle internalization via magnetic compulsion through the cell membrane. Additionally, Fe-MOFs may exert magnetic-triggered hyperthermia or magnetothermal effects that further contribute to cell death.^51^ Albeit hyperthermia was not directly investigated in the current study, strong cytotoxicity enhancement in the presence of the magnet at the higher doses and longer periods offers that cellular uptake and cell death can be magnetically induced under certain conditions.

### 3.4 Effects of Fe-MOF and DOX Cotreatment on Cell Viability

The therapeutic potential of the synthesized Fe-MOF samples was evaluated as combined treatment. Hence, a well-known chemotherapy agent, DOX, was loaded onto the constructs and tested in both cell lines under varying concentrations, exposure times, and magnetic field conditions, clearly demonstrating a varying synergistic interaction between DOX and Fe-MOF carriers according to the distinct combinations of MOF composition, exposure duration, and magnetic stimulation (Figure 9).

**Figure 9:**
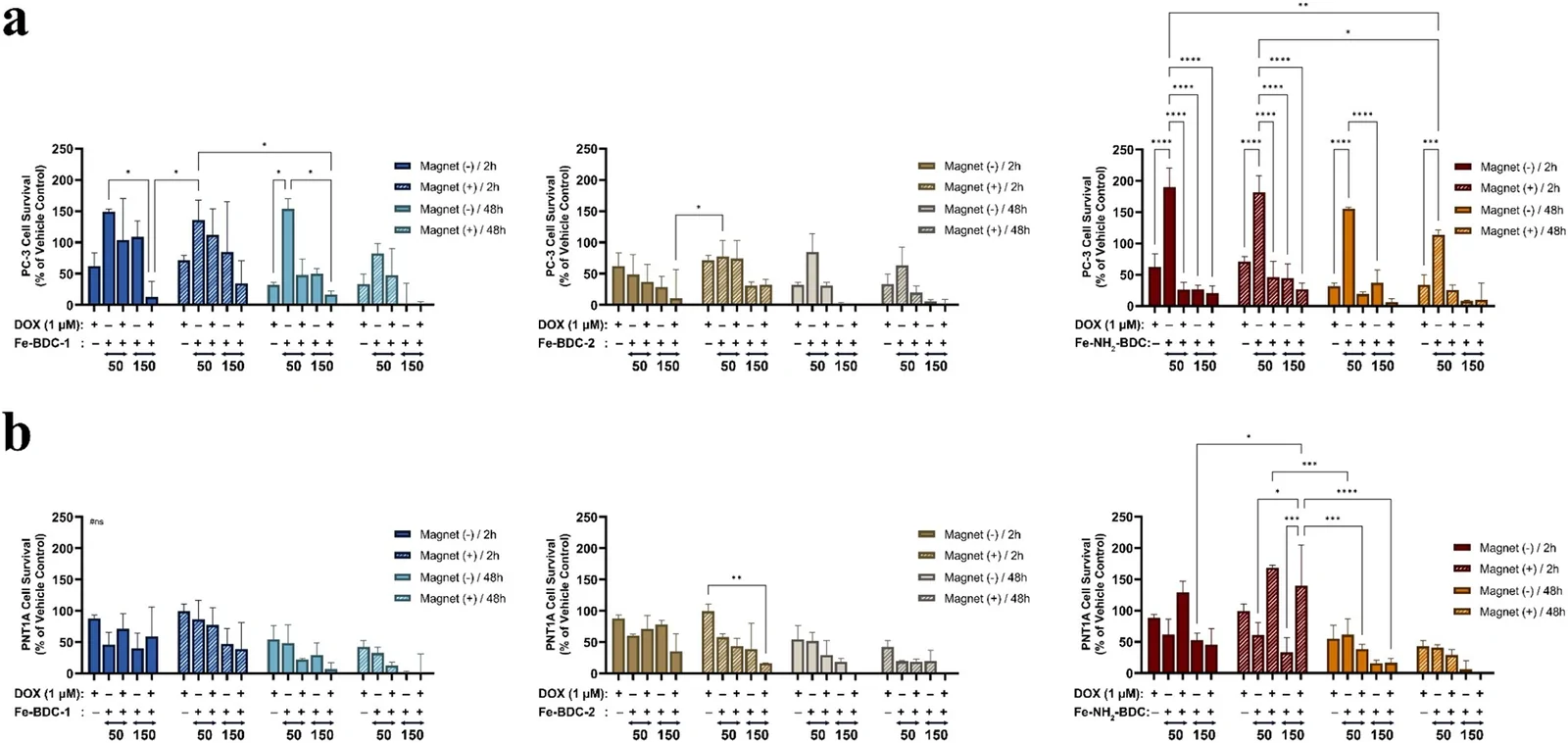
Assessment of cytotoxic effects of DOX-loaded Fe-BDC-1 (left panels), Fe-BDC-2 (middle panels), and Fe-NH_2_-BDC (right panels) in the **a)** PC-3 and **b)** PNT1A cell lines in the absence and presence of the magnet. Statistical analysis (mean ± SD, n = 3 independent experiments) was performed using two-way ANOVA (*p ≤ 0.05, **p ≤ 0.01, ***p ≤ 0.001, ****p ≤ 0.0001).

Although DOX-loaded Fe-BDC-1 (50 μg/mL) did not exhibit cytotoxicity on PC-3 cells at early and late time points compared to DOX only, DOX loading appeared to improve Fe-BDC-1 effects relative to Fe-BDC-1 (50 μg/mL) only (Figure 9a, left). These observations for the lower Fe-BDC-1 concentration emerged either under magnetic or magnet-free conditions. Contrariwise, PC-3 viability diminished by 5.1 and 2.1 folds upon DOX-loaded Fe-BDC-1 (150 μg/mL) treatment for 2 h relative to only DOX administration under magnet (−) and magnet (+) conditions, respectively. This DOX-improved cytotoxicity in PC-3 cells was more striking when compared to Fe-BDC-1 (150 μg/mL) treatment (Figure 9a, left). However, PC-3 viability remained relatively lower at 48 h according to the DOX-only and Fe-BDC-1 (150 μg/mL)-only groups. Surprisingly, DOX with low-concentration Fe-BDC-1 (50 μg/mL) at both time points and high-dose Fe-BDC-1 (150 μg/mL) at 2 h did not cause a significant alteration in PNT1A survival (Figure 9b, left); however, the response of Fe-BDC-1 (150 μg/mL)+DOX at 48 h resembled the cytotoxicity profile in PC-3 (Figure 9, left panels). Given these data, synergistic effects between DOX and Fe-BDC-1 were more evident at higher doses.

DOX-loaded Fe-BDC-2 roughly demonstrated better cytotoxicity on the PC-3 cell line across all conditions. DOX loading on Fe-BDC-2 (50 μg/mL) had obviously no or only a limited impact (up to ∼10%) at 2 h under both magnet-related conditions (Figure 9a, middle). At 48 h, PC-3 viability upon Fe-BDC-2 (150 μg/mL)+DOX cotreatment approached complete cell death under the magnet-free state, whereas Fe-BDC-2 (50 μg/mL)+DOX could alleviate the PC-3 viability by approximately 63% according to Fe-BDC-2 (50 μg/mL)-only under the same condition. Intriguingly, magnetic exposure seemed to further enhance the cytotoxic effect of DOX application with even 50 μg/mL of Fe-BDC-2 at 48 h, reducing PC-3 viability by 69% and 41.1% relative to Fe-BDC-2 (50 μg/mL) and DOX only, respectively (Figure 9a, middle). Furthermore, similar results were detected at the higher concentration of Fe-BDC-2, suggesting that DOX and Fe-BDC-2 may have a better synergistic interaction between the carrier system and the chemotherapeutic agent than Fe-BDC-1 in malignant PC-3 cells. Otherwise, PNT1A cells were slightly shielded from DOX alone, Fe-BDC-2 alone (either lower or higher), and DOX + Fe-BDC-2 (either lower or higher) combinations at the early time point (Figure 9b, middle). Nevertheless, treatment with DOX + Fe-BDC-2 together showed an eradicating response at 48 h (Figure 9b) just like in PC-3 (Figure 9, middle panels).

Among all formulations, Fe-NH_2_-BDC+DOX combination represented the most consistent and potent cytotoxic activity on the PC-3 cell line. At 2 h, low-dose Fe-NH_2_-BDC treatment (50 μg/mL) could not suppress the cell survival, while DOX alone resulted in a 30–40% decline at both magnetic states (Figure 9a, right). Notably, DOX treatment in combination with the low-concentration Fe-NH_2_-BDC led to about a 2.4-fold and 1.5-fold reduction compared to DOX alone under the magnet (−) and magnet (+) conditions, respectively. DOX had a negligible improvement in PC-3 cytotoxicity in the company with high-dose Fe-NH_2_-BDC (150 μg/mL) treatment for 2 h relative to Fe-NH_2_-BDC (150 μg/mL) only without the magnetic field; however, DOX-loaded Fe-NH_2_-BDC (150 μg/mL) could enhance cytotoxicity up to 1.7-fold under the magnet (+) condition. Meanwhile, when DOX-loaded Fe-NH_2_-BDC (150 μg/mL) was compared to DOX alone at 2 h, synergistic effects were considerably more obvious, leading to 3.1-fold and 2.7-fold downward trends in the state of the magnet (−) and the magnet (+), respectively. Similarly, high-dose Fe-NH_2_-BDC alone (150 μg/mL) also had no cytotoxic effect on PC-3 cells at 48 h, while DOX alone decreased cell survival approximately 70% according to the control (Figure 9a, right). Besides, prolonged exposure time (48 h) to the compounds dramatically elevated cytotoxicity, particularly at high-dose Fe-NH_2_-BDC. Co-treatment of DOX and Fe-NH_2_-BDC (150 μg/mL) resulted in a 3.5-fold and 5.1-fold upgrade in cytotoxic responses relative to DOX-only under magnetic exposure and without the magnet, respectively (Figure 9a, right). Similar to other Fe-MOF samples, PNT1A cells were relatively resistant to all Fe-BDC-2+DOX combinations at 2 h, so much so that no retardation has been designated in PNT1A viability under the magnetic field (Figure 9b, middle). However, compounds alone or in combination emerged with similar cytotoxicity profiles at 48 h in both malignant and nonmalignant cell lines (Figure 9, right panels).

These exhaustive insights collectively suggest that DOX-loaded Fe-MOF constructs may exert their antitumor activity through a multifactorial and condition-dependent synergistic mechanism, in which the intrinsic properties of the MOF scaffold, drug loading efficiency, exposure time, and magnetic modulation collectively determine the final biological outcome. Among the tested samples, Fe-BDC-2 and particularly Fe-NH_2_-BDC demonstrated a favorable synergy with DOX compared to Fe-MOFs or DOX alone, highlighting the importance of framework structure and surface functionalization in dictating therapeutic potential. Herein, cumulative evidence has reported that amine functionalization on certain iron- or zirconium-based MOFs is suitable for anticancer DOX delivery due to favorable physicochemical interactions between MOFs and drugs, increased cellular uptake, targeted drug distribution, tunable spatiotemporal drug release kinetics, and enhanced tumor eradication efficacy.^44,45,53,54^ Consequently, Fe-NH_2_-BDC represents a consistency with the previous data, standing as a promising candidate for translational studies.

### 3.5 Flow-Cytometric Characterization of Treatment-Associated Cell-Death Patterns

As elaborated so far, Fe-NH_2_-BDC has come out as more conceivable across the time points, magnetic conditions, and DOX co-treatment. Hence, we delved into the cytotoxic effects of Fe-NH_2_-BDC in PC-3 cells at 48 h, which yielded deducible and consistent outcomes, to gain insights about its mechanism of action. Annexin V/Propodium Iodide (PI) staining was performed 48 h exposure of Fe-NH_2_-BDC alone or in combination with DOX under the magnet-free condition (Figure 10).

**Figure 10:**
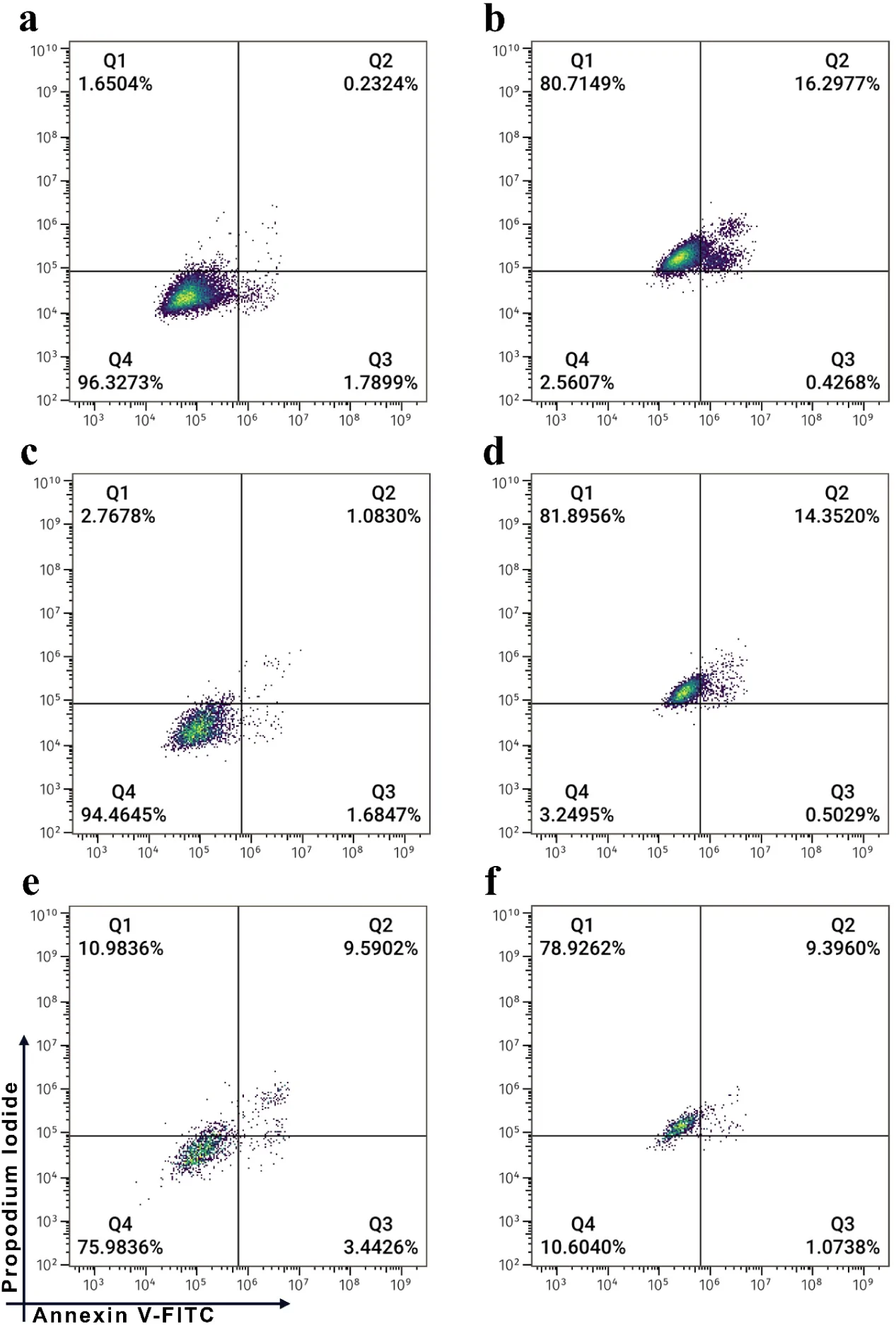
Flow cytometric analysis of cell death mechanisms in PC-3 cells following 48 h treatment with **a)** vehicle control, **b)** DOX only, **c)** Fe-NH_2_-BDC (50 μg/mL), **d)** Fe-NH_2_-BDC (50 μg/mL)+DOX, **e)** Fe-NH_2_-BDC (150 μg/mL), and **f)** Fe-NH_2_-BDC (150 μg/mL)+DOX. Quadrants Q4, Q3, Q2, and Q1 represent viable, early apoptotic, late apoptotic, and necrotic cell fractions, respectively.

In the vehicle control (Figure 10a), the vast majority of PC-3 cells were viable (96.3%), confirming the physiological integrity of untreated cells. Meanwhile, the predominant population shifted toward the PI^+^/Annexin V^–^ area (Q1:80.7%), whereas early apoptotic (Q3) and late apoptotic (Q2:16.3%) fractions remained relatively limited (Figure 10b). This distribution profile suggests that DOX (1 μM) primarily triggered a necrotic phenotype instead of apoptosis in the PC-3 cell line under the current experimental conditions. Albeit DOX is conventionally known as an apoptosis-causing chemotherapeutic agent through p53-dependent signaling pathways, several studies have reported that PC-3 cells possess resistance to apoptosis due to their androgen-independence, p53-null status, and impaired apoptotic execution,^55–57^ thereby shifting DOX-mediated death toward necrosis or necroptosis rather than canonical apoptosis. Moreover, we also encountered a noteworthy debris fraction during the post-flow cytometric analysis, offering excessive ROS generation by DOX could have elicited membrane rupture and necrotic collapse instead of mitochondria-dependent apoptotic progression.

Treatment with Fe-NH_2_-BDC alone at 50 μg/mL disclosed approximately 94.5% viable cells and caused only minimal apoptosis or necrosis (Figure 10c), highly comparable with the previous data (Figures 7a–9a), verifying that low-dose Fe-NH_2_-BDC alone exerted only restricted cytotoxicity toward PC-3 cells at 48 h. In contrast, co-treatment with Fe-NH_2_-BDC (50 μg/mL) and DOX preserved the overall necrotic tendency (Figure 10d). Nevertheless, considering the cytotoxicity assays (Figures 7a–9a), Fe-NH_2_-BDC at 50 μg/mL enhanced the cytotoxic performance of DOX compared to either agent alone. Given these findings, low-concentration Fe-NH_2_-BDC likely improved intracellular drug accumulation and/or therapeutic potential, although it could mainly function as a carrier without substantially modifying the end point death mechanism induced by DOX alone.

Fe-NH_2_-BDC at the higher concentration (150 μg/mL) alone increased both apoptotic and necrotic populations compared to its lower concentration. The viable cell fraction declined to 76%, while late apoptotic, early apoptotic, and necrotic populations raised to nearly 9.6%, 3.4%, and 11%, respectively (Figure 10e), importantly highlighting that the previously observed cytotoxicity (Figure 7a–9a) involved a mixed cell death phenotype incorporating both apoptosis and necrosis. Notably, co-treatment with Fe-NH_2_-BDC (150 μg/mL) and DOX dramatically altered the cell death landscape (Figure 10f). The viable population declined to 10.6%, while necrotic (∼79%) and apoptotic (9.4%) cells became relatively apparent. Moreover, an outstanding debris fraction was also prevailing on the FCS/SSC plot of both groups, altogether indicating loss of membrane integrity in Fe-NH_2_-BDC-treated PC-3 cells alone or in combination with DOX.

The Annexin V/PI results do not demonstrate that Fe-NH_2_-BDC co-treatment enhanced DOX-induced apoptosis. Under the tested conditions, the DOX-alone group exhibited a higher Annexin V-positive apoptotic fraction, whereas the high-dose Fe-NH_2_-BDC+DOX group was characterized predominantly by PI-positive membrane-compromised cells. This observation indicates a shift in the distribution of cell-death phenotypes rather than an increase in apoptosis. The apparent difference from the WST-1 results should also be interpreted in view of the different assay end points: WST-1 measures metabolic activity, whereas Annexin V/PI analysis distinguishes membrane integrity and phosphatidylserine exposure. Accordingly, the flow-cytometric data are presented as descriptive mechanistic observations and do not establish superiority of the combination over DOX alone.

In addition, the flow-cytometric samples were obtained by pooling duplicate wells and were not evaluated as independent biological replicates; therefore, the reported population percentages should not be interpreted as statistically validated differences between treatments.

Collectively, Annexin V/PI analysis strongly supports the cytotoxicity data, unveiling the varying modes of cell death according to the treatment dose and combination. While low-dose Fe-NH_2_-BDC relatively acts as a biocompatible drug carrier, the higher concentration, particularly in combination with DOX, promotes extensive membrane damage and a mixed apoptotic/necrotic phenotype, probably driven by amplified oxidative stress and iron-dependent cytotoxicity. Recent literature increasingly supports the concept that Fe-based MOFs can induce mixed apoptotic-necrotic or ferroptotic responses depending on dosage, degradation kinetics, and intracellular iron release profiles.^58–62^ Particularly, Fe-based nanomaterials have been shown to trigger ferroptosis by releasing reDOX-active iron species, promoting Fenton-like reactions, and enhancing ROS generation.^61,63,64^ On the other hand, ferroptosis generally exhibits an Annexin V^–^/membrane-impermeant dye^+^ or double positive profile.^65–67^ In this regard, the stronger cytotoxic responses detected for Fe-BDC-2 and Fe-NH_2_-BDC under different combinatorial conditions (magnet–/+ and/or DOX −/+) so far may partially involve labile ferroptosis-related mechanisms in addition to conventional apoptotic or necrotic pathways, which remains a matter that needs to be clarified as a central limitation of the current study. These findings altogether reinforce the translational potential of Fe-MOFs as a multifaceted therapeutic nanoplatform, combining intrinsic anticancer activity and chemotherapy delivery.

## 4 Conclusion

This study demonstrates that both Fe precursor selection and ligand functionalization decisively modulate the structure–property–bioactivity relationship of Fe-based MOFs. Fe-BDC-1 and Fe-BDC-2, prepared with the same BDC linker, shared a related framework chemistry, MIL53­(Fe)-*like* structure, but FeCl_3_ produced a more crystalline Fe-BDC material with a distinct morphology and stronger intrinsic cytotoxicity toward PC-3 prostate cancer cells. Replacing BDC with NH_2_-BDC generated a separate amino-functionalized Fe-MOF phase, assigned tentatively to an MIL-88B­(Fe)-*like structure*, with altered morphology, expanded visible absorption, reduced band-gap energy, and strong photoluminescence. The biological data show that Fe-MOF activity is not governed by composition alone but by the combined effects of framework structure, concentration, exposure time, magnetic stimulation, and DOX co-treatment. Fe-BDC-2 was the most intrinsically cytotoxic scaffold in PC-3 cells. In the WST-1 viability assays, Fe-NH_2_-BDC showed the most consistent interaction with DOX under selected conditions. Nevertheless, the flow-cytometric findings did not demonstrate enhancement of DOX-induced apoptosis and instead indicated a predominantly necrotic or membrane-compromised phenotype. These findings should therefore be regarded as preliminary evidence of a condition-dependent cytotoxic interaction rather than proof of synergistic drug delivery or enhanced apoptotic efficacy. Accordingly, Fe-NH_2_-BDC represents a candidate for further mechanistic and drug-delivery investigations rather than an established multifunctional anticancer platform. Future studies should quantify DOX loading and release, intracellular uptake, ROS generation, ferroptosis-related markers, and in vivo tumor selectivity to clarify the mechanism and translational potential of this system.

## Data Availability

The data that support the findings of this study are available from the corresponding author upon reasonable request.
